# Methodological Insights Into T‐Cell Activation: CD3/CD28 Versus PMA/Ionomycin Stimulation

**DOI:** 10.1002/eji.70227

**Published:** 2026-06-17

**Authors:** Nekruz Abdulhaqov, Lady Tatiana Albarracin Melo, Ines Meinert, Stephan Fricke, Luca Simeoni

**Affiliations:** ^1^ Medical Faculty Institute for Clinical Immunology and Cell Therapeutics, Otto‐von‐Guericke University Magdeburg Germany; ^2^ Republican Oncology Research Center I. Somoni Avenue Dushanbe Tajikistan; ^3^ Health Campus Immunology, Infectiology and Inflammation (GC‐I^3^), University Hospital, Otto‐von‐Guericke University Magdeburg Germany; ^4^ Center for Health and Medical Prevention (CHaMP), University Hospital Otto‐von‐Guericke University Magdeburg Germany; ^5^ Fraunhofer Institute for Cell Therapy and Immunology Leipzig Germany

## Abstract

PMA/ionomycin and CD3/CD28 stimulation induce fundamentally distinct signaling programs in primary human T cells. While CD3/CD28 stimulation activates both ERK and IL‐2/STAT5 signaling pathways, PMA/ionomycin induces predominantly ERK‐dependent proliferation despite robust IL‐2 production and CD25 upregulation. These findings highlight important mechanistic and methodological differences between pharmacological and receptor‐mediated T‐cell activation.

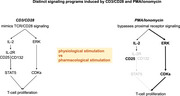

T‐cell activation is essential for adaptive immune responses [1]. Engagement of the T‐cell receptor (TCR) complex together with costimulatory receptors such as CD28 initiates signaling cascades that control cytokine production, clonal expansion, and differentiation. In vitro, this process is commonly mimicked by stimulating T cells with anti‐CD3 and anti‐CD28 antibodies [2]. In parallel, pharmacological stimulation with phorbol 12‐myristate 13‐acetate (PMA) and the calcium ionophore ionomycin is also widely used [3].

As both methods induce T‐cell activation, they are often considered interchangeable. However, a recent transcriptomic study demonstrated that PMA/ionomycin elicits a transcriptional profile distinct from TCR‐mediated activation, suggesting substantial mechanistic divergence [4]. The molecular basis underlying these differences, however, remains poorly understood.

Here, we performed a side‐by‐side molecular comparison of signaling pathways and activation events in human peripheral blood T cells stimulated via CD3/CD28 antibodies versus PMA/ionomycin.

It is well established that PMA/ionomycin stimulation strongly induces IL‐2 production as well as upregulation of the IL‐2 receptor (IL‐2R) α chain (CD25). Indeed, IL‐2 production and CD25 expression are significantly higher in primary human T cells stimulated with PMA/ionomycin compared to CD3/CD28 stimulation after 24 h (Figure 1A,B). We next examined STAT5 phosphorylation, a key downstream event of IL‐2R signaling that is essential for T‐cell proliferation [5]. Despite robust IL‐2 production and CD25 upregulation, PMA/ionomycin stimulation induced only weak STAT5 phosphorylation across all time points (6, 12, and 24 h post‐stimulation), whereas CD3/CD28 stimulation resulted in strong STAT5 activation (Figure 1C). In contrast, phosphorylation of ERK1/2 was readily induced under both stimulation conditions (Figure 1C). Addition of PMA to CD3/CD28‐stimulated T cells prevented STAT5 phosphorylation, suggesting that pharmacological stimulation interferes with IL‐2R signaling (Figure 1C).

**FIGURE 1:**
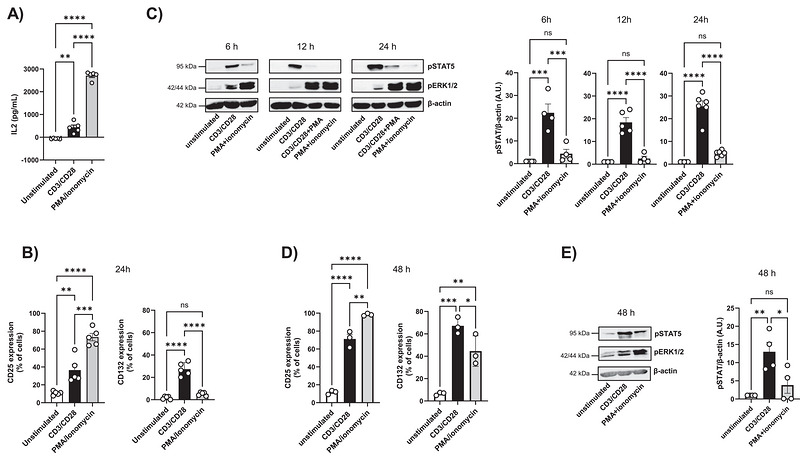
Defective activation of the IL‐2/IL‐2R signaling axis upon PMA/ionomycin stimulation. Human peripheral blood T cells were stimulated with plate‐bound CD3/CD28 antibodies or PMA/ionomycin. (A) IL‐2 production was analyzed by ELISA. (B and D) Expression of IL‐2 receptor chains was assessed by flow cytometry after 24 h (B) or 48 h (D). (C and E) Phosphorylation of STAT5 and ERK1/2 was analyzed by immunoblotting after stimulation for the indicated time points (C) or for 48 h (E). One representative experiment is shown. Graphs represent quantification of immunoblots normalized to β‐actin. Statistical analyses were performed using one‐way ANOVA with Tukey's multiple comparison test; ****p < 0.0001, ***p < 0.001, **p < 0.01, *p < 0.05. PMA, phorbol 12‐myristate 13‐acetate.

To assess whether signaling strength influences pathway activation, we titrated anti‐CD3/CD28 stimulation using antibody‐coated beads. Both STAT5 and ERK1/2 phosphorylation were consistently induced across all conditions tested (Figure S1A). In contrast, stimulation with soluble anti‐CD3 and anti‐CD28 antibodies did not induce sustained STAT5 or ERK1/2 phosphorylation at later time points (Figure S1A), consistent with previous reports showing that soluble antibodies induce only transient signaling without productive T‐cell activation [6, 7].

Although PMA/ionomycin strongly upregulated CD25 and induced high IL‐2 secretion, STAT5 phosphorylation remained weak. Therefore, we examined the surface expression of CD132 (common γ chain) and CD122 (β chain), which, together with CD25, form the high‐affinity IL‐2R. Lee et al. reported that PMA/ionomycin failed to induce CD132 at a transcriptional level [4]. We therefore hypothesize that defective signaling results from impaired CD132 expression and consequently defective IL‐2R assembly. Indeed, we found that PMA/ionomycin failed to induce robust CD132 upregulation in primary human T cells 24 h after stimulation, whereas CD3/CD28 stimulation did so (Figure 1B). Thus, although PMA/ionomycin promotes IL‐2 secretion and CD25 expression, it concurrently failed to induce CD132 transcription and surface expression, preventing the formation of a functional high‐affinity IL‐2R and efficient downstream signaling. On the other hand, CD122 expression was barely detectable in both resting and activated T cells (data not shown), consistent with previous reports [8, 9, 10].

We next examined whether CD132 expression is delayed upon PMA/ionomycin stimulation. PMA/ionomycin induced partial CD132 upregulation at 48 h, although still substantially lower than after CD3/CD28 stimulation (Figure 1D), whereas CD25 expression was robustly induced under both stimulation conditions (Figure 1D). STAT5 phosphorylation remained weak upon PMA/ionomycin stimulation (Figure 1E), consistent with delayed and incomplete formation of the high‐affinity IL‐2 receptor complex.

We next assessed T‐cell proliferation. As shown in Figure 2A,B, both CD3/CD28 and PMA/ionomycin induced robust proliferation. CD3/CD28‐driven proliferation was sensitive to neutralizing antibodies against IL‐2, JAK inhibition, and MEK1/2 inhibition, whereas PMA/ionomycin‐driven proliferation was resistant to IL‐2 neutralization or JAK inhibition, but remained sensitive to MEK1/2 inhibition (Figure 2A,B). Similar sensitivity to MEK1/2 inhibition was observed in purified naïve CD4^+^, memory CD4^+^, and CD8^+^ T‐cell subsets (Figure S1B).

**FIGURE 2:**
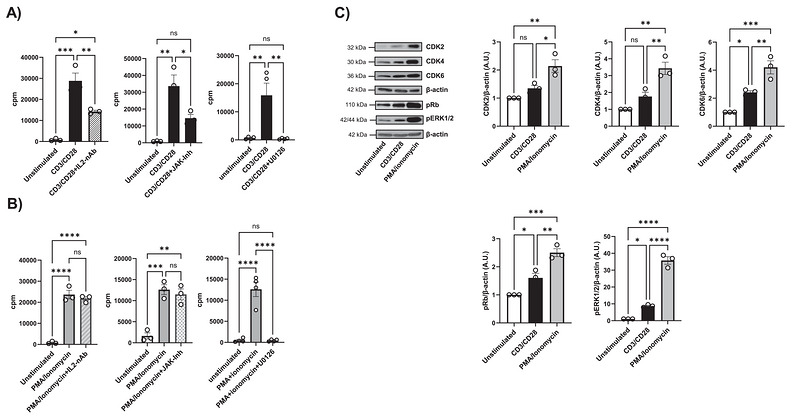
IL‐2‐signaling‐independent proliferation upon PMA/ionomycin stimulation. Human peripheral blood T cells were stimulated with CD3/CD28 antibodies (A) or PMA/ionomycin (B) in the presence or absence of IL‐2‐neutralizing antibody, JAK inhibitor, or MEK1/2 inhibitor (U0126). Proliferation was analyzed after stimulation. (C) Expression of CDK2, CDK4, CDK6, phosphorylated Rb, and phosphorylated ERK1/2 was assessed by immunoblotting after 24 h stimulation. One representative experiment is shown. Graphs represent quantification normalized to β‐actin. Statistical analyses were performed using one‐way ANOVA with Tukey's multiple comparison test; ****p < 0.0001, ***p < 0.001, **p < 0.01, *p < 0.05. PMA, phorbol 12‐myristate 13‐acetate.

Together, these findings indicate that PMA/ionomycin‐induced proliferation primarily depends on ERK1/2 signaling and occurs largely independently of IL‐2/STAT5 signaling. This uncoupling of proliferation from canonical IL‐2R/STAT5 signaling represents a major difference between pharmacological and receptor‐mediated T‐cell activation.

To investigate the mechanism, we assessed the expression of cell cycle regulators. PMA/ionomycin strongly induced the phosphorylation of ERK1/2 and retinoblastoma protein (Rb) and increased the expression of cyclin‐dependent kinases (CDK2, CDK4, and CDK6) (Figure 2C), suggesting direct activation of the Ras/MAPK/CDK axis [11]. These findings provide a mechanistic explanation for IL‐2‐independent proliferation upon pharmacological stimulation.

Our findings demonstrate that CD3/CD28 and PMA/ionomycin stimulate T cells through fundamentally distinct mechanisms. CD3/CD28 engages both the JAK/STAT and Ras/ERK pathways and relies on IL‐2 signaling for proliferation. In contrast, PMA/ionomycin bypasses proximal receptor signaling and promotes proliferation predominantly through ERK/CDK signaling.

A recent study demonstrated that TCR/CD28 stimulation upregulates CD132 expression in murine lymph node T cells [12], corroborating our findings and providing clear evidence that the γ‐chain is regulated in a TCR signaling‐dependent manner.

Although delayed and partial upregulation of CD132 may contribute to impaired STAT5 activation, we cannot exclude the possibility that PMA/ionomycin additionally interferes with JAK/STAT signaling downstream of the IL‐2 receptor, for example via PKC‐dependent mechanisms. Indeed, PMA has been reported to inhibit IL‐6‐induced STAT3 phosphorylation, suggesting that PKC‐driven signaling can antagonize JAK/STAT pathways [13].

In conclusion, CD3/CD28 and PMA/ionomycin engage divergent signaling mechanisms. Despite robust IL‐2 production and CD25 upregulation, PMA/ionomycin induced only weak activation of the IL‐2/IL‐2R/STAT5 axis and promoted T‐cell proliferation predominantly through ERK1/2 signaling. These findings provide methodological guidance for T‐cell activation studies and demonstrate that IL‐2 production and CD25 upregulation do not necessarily reflect functional IL‐2R/STAT5 signaling.

## Author Contributions

**Nekruz Abdulhaqov**: data curation, formal analysis, investigation, methodology, validation, visualization. **Lady Tatiana Albarracin Melo**: data curation, formal analysis, investigation, methodology. **Ines Meinert**: investigation, methodology. **Stephan Fricke**: funding acquisition, project administration, resources, supervision. **Luca Simeoni**: conceptualization, funding acquisition, methodology, project administration, resources, supervision, visualization, writing – original draft, writing – review and editing.

## Funding

This research was supported by the state of Saxony‐Anhalt (European Regional Development Fund, Autonomy in Aging, INITIATE) and by funding of the Deutsche Forschungsgemeinschaft (DFG) (SI 861/4‐1) to Luca Simeoni. We acknowledge financial support for consumables from the “Masterstudiengang Immunologie” of the Medical Faculty of the Otto‐von‐Guericke University, provided in the context of the Master's thesis of Nekruz Abdulhaqov, first author of this manuscript. Lady Tatiana Albarracin Melo was additionally supported by a scholarship from the Medical Faculty of the Otto‐von‐Guericke University.

## Conflicts of Interest

The authors declare no conflicts of interest.

## Supporting information




**Supporting File 1**: eji70227‐sup‐0001‐SuppMat.pdf.

**Supporting File 2**: eji70227‐sup‐0002‐Material_Methods.docx.

## Data Availability

The data that support the findings of this study are available from the corresponding author upon request.
