# Supplementary material for: Methodological Insights Into T‐Cell Activation: CD3/CD28 Versus PMA/Ionomycin Stimulation
Source: Eur J Immunol. 2026 Jun 17;56(6):e70227. doi: 10.1002/eji.70227 (PMC13273920; doi:10.1002/eji.70227)
Supplement: Supplementary file 1 — Supporting File 1: eji70227‐sup‐0001‐SuppMat.pdf. [file EJI-56-e70227-s001.pdf]

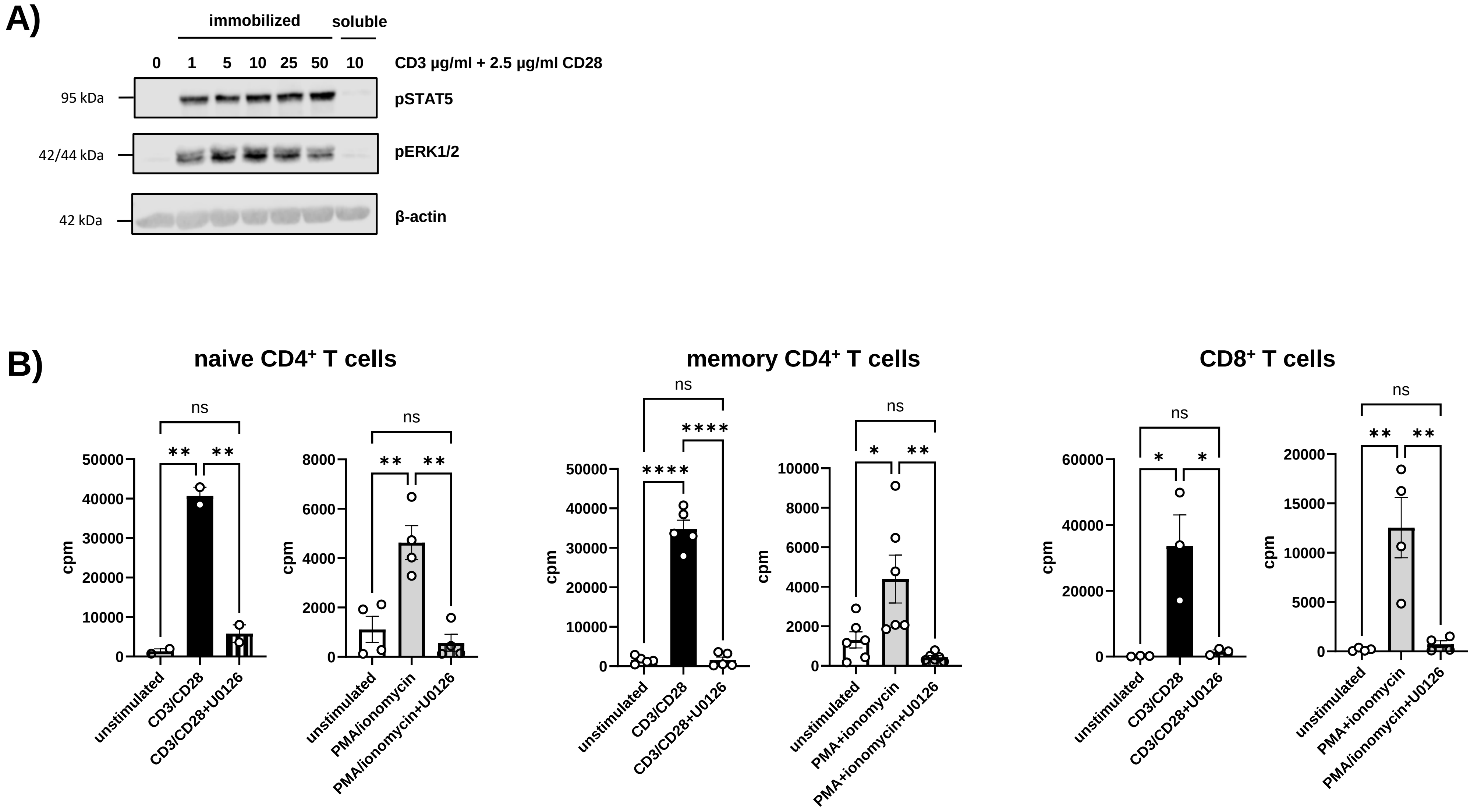

Supplementary Figure 1. **A)** Peripheral blood T cells were stimulated with different amount of CD3 and CD28 (2.5 µg/ml) antibodies coated on microbeads or with 10 µg/ml CD3 and 2.5 µg/ml CD28 applied in solution for 6 hours. After stimulation, cell lysates were assayed for the phosphorylation of STAT5 (pSTAT5) and Erk1/2 (pERK1/2), as well as β-actin as loading control. One representative experiment of four is shown. **B)** Peripheral blood T cells were stimulated with plate-bound CD3 (10 µg/ml) and CD28 (2.5 µg/ml) antibodies or 20 ng/ml PMA and 0.4 µg/ml ionomycin. Proliferation of the stimulated cells is shown in the presence or absence of 10µM U0126.
