# Supplementary material for: Methodological Insights Into T‐Cell Activation: CD3/CD28 Versus PMA/Ionomycin Stimulation
Source: Eur J Immunol. 2026 Jun 17;56(6):e70227. doi: 10.1002/eji.70227 (PMC13273920; doi:10.1002/eji.70227)
Supplement: Supplementary file 2 — Supporting File 2: eji70227‐sup‐0002‐Material_Methods.docx. [file EJI-56-e70227-s002.docx]

**­­Materials and Methods**

**T-cell isolation and cultivation**

Peripheral blood was collected from healthy donors at the Institute of Molecular and Clinical Immunology with ethical approval (Ethics Committee OvGU, 107/09), and all blood donors provided written informed consent. Isolation of human T-cells was performed on the day of blood collection via a three-step protocol.

*PBMC isolation:*

Whole blood was anticoagulated immediately with heparin (Biochrom) (5 U/ml) in a 2:1 ratio. Subsequently, 35 mL of heparinized blood was carefully layered over 15 mL of Pancoll human (PAN Biotech, Aidenbach, Germany) density gradient medium in 50 mL tubes and centrifuged at 400 × g for 30 min at room temperature without brake. The interphase containing peripheral blood mononuclear cells (PBMCs) was collected and washed three times with RPMI 1640 medium (400 × g, room temperature).

*Magnetic labeling and separation:*

PBMCs were counted and labeled using the T Cell Isolation Kits (Miltenyi Biotec GmbH, Bergisch Gladbach, Germany) according to the manufacturer’s protocol. Cells were incubated sequentially with biotinylated antibodies cocktail MicroBeads at 2–8°C, followed by magnetic separation using LS columns in a MACS separator under the "Depletes" program. The unlabeled flow-through fraction enriched for untouched T cells was collected.

*Cell culture:*

Isolated T-cells were cultured in RPMI 1640 supplemented with 10% fetal bovine serum (FBS, Sigma-Aldrich) and 2 μg/ml ciprofloxacin (Fresenius Kabi Austria GmbH) at 37°C, 5% CO₂ until further use.

**T-cell stimulation**

T cells were stimulated via two methods: (1) CD3 (10 µg/mL, UHCT1, Biolegend) CD28 (2.5 µg/mL, CD28.2, Biolegend) antibodies precoated overnight at 4°C on 12-well plates, and (2) pharmacological mitogens PMA (20 ng/mL, Sigma Aldrich) plus ionomycin (0.4 µg/mL, Sigma Aldrich). After washing off unbound antibodies, 1.5 × 10⁶ cells/mL were cultured at 37°C for 12 or 24 hours for analyses.

**T-cell proliferation assay**

Proliferation was measured by [³H]-thymidine incorporation. T cells were stimulated in 96-well plates with anti-CD3/CD28 antibodies or PMA/ionomycin, with or without IL-2 neutralizing antibody (0.2 µg/mL, 5334, BD), a JAK inhibitor (0.25 µM, STEMCELL), or MEK1/2 inhibitor U0126 (10µM, Calbiochem). T cells were incubated at 37°C, 5% CO₂ for 72 h. [³H]-thymidine was added at 0.2µCi/well for the final 6–8 h, followed by harvesting on glass fiber filters and scintillation counting of incorporated radioactivity.

**Cell lysis**

Cells (4.5 × 10⁶) were lysed in lysis buffer (1% lauryl maltoside, 1% NP-40, 1 mM phenylmethylsulphonyl fluoride, 1mM Na3VO4, 10 mM NaF, 10 mM EDTA, 50 mM Tris–HCl pH 7.5, and 150 mM NaCl) on ice for 20 min. After centrifuged at 16,000 × g, 4°C for 10 min, supernatants were mixed with 5× sample buffer (Tris pH 6.8 100mM, Glycerol 50%, Bromophenol Blue 0.25%, SDS 5%, and DTT), heated at 99°C for 5 min, and stored for western blot analyses.

**Western blot**

Proteins were separated by SDS-PAGE using 10% separating gel, transferred semi-dry onto nitrocellulose membranes, and blocked with 5% milk in TBS (Tris-buffered saline)/ 0.1% Tween 20. Membranes were incubated with primary antibodies (1 h, RT) in 5% bovine serum albumin (BSA) in TBS/0.1% Tween 20, washed and incubated with fluorophore-conjugated secondary antibodies diluted in 5% milk or TBS 1 h at RT. Proteins were detected using Odyssey LI-COR imaging. Protein bands were quantified by Image Studio, normalized to loading controls. The following antibodies were used: pT202/Y204 Erk1/2 (Cat. #9101S), pY694 STAT5 (Cat. #2793273), pS780 Rb (Cat. #9307S), CDK2 (clone 78B2), CDK4 (clone D9G3E), CDK6 (clone DCS83) from Cell Signaling Technology, and β-actin (clone AC-15) from Sigma-Aldrich.

**ELISA**
IL-2 secretion was quantified by sandwich ELISA (R&D Systems) using monoclonal capture antibodies coated on microplates, with detection via peroxidase-conjugated secondary antibodies and TMB substrate. Absorbance was measured and IL-2 concentration interpolated from a standard curve.

**Flow cytometry**

T cells (1 × 10⁶/mL) were stained for surface markers CD25, CD122, and CD132 with fluorochrome-conjugated antibodies anti-CD25-PE (M-A251, BD Biosciences), anti-CD122 -APC (TUGh4, Biolegend), anti-CD132-APC (Mik-β3, BD Pharmingen) at 4°C in the dark for 20 min. After washing, cells were analyzed using a LSRFortessa I (BD Biosciences). Data were processed with FlowJo software.

**Statistics**
Data visualization and statistics were conducted with GraphPad Prism 10. Statistical significance was assessed using a One-Way ANOVA, Tukey's multiple comparisons test, ****p≤0.0001, ***p≤0.001, **p≤0.01, *p≤0.05.
